# A Leaf-Expressed *TERMINAL FLOWER1* Homolog from Coffee with Alternative Splice Forms Alters Flowering and Branching in Arabidopsis

**DOI:** 10.3390/plants15142162

**Published:** 2026-07-14

**Authors:** Carlos Henrique Cardon, Victoria Lesy, Catherine Fust, Thales Henrique Cherubino Ribeiro, Owen Hebb, Raphael Ricon de Oliveira, Mark A. A. Minow, Gabriel de Campos Rume, Antonio Chalfun-Junior, Joseph Colasanti

**Affiliations:** 1 Department of Molecular and Cellular Biology, University of Guelph, Guelph, ON N1G 2W1, Canada; victoria.lesy@gmail.com (V.L.); cfust@uoguelph.ca (C.F.); owen.hebb@gmail.com (O.H.); mark.minow@uga.edu (M.A.A.M.); 2 Laboratory of Plant Physiology and Molecular Biology, Plant Physiology Sector, Department of Biology, Federal University of Lavras, Lavras 37203-202, MG, Brazil; thalescherubino@gmail.com (T.H.C.R.); rapharicon@gmail.com (R.R.d.O.); gabriel.rume@gmail.com (G.d.C.R.)

**Keywords:** flowering time, coffee flowering, perennial plants, floral repressor, homologs, alternative splicing, transcription complexes, reproductive development

## Abstract

**Coffee** is a perennial plant that exhibits asynchronous flowering while maintaining concomitant vegetative growth. This growth dichotomy affects fruit development and maturation time. To better understand flowering in coffee, we characterized a phosphatidylethanolamine binding protein (PEBP) homolog with high similarity to *Arabidopsis thaliana TERMINAL FLOWER1* (*TFL1*). The interaction of TFL1 with floral regulator bZIP transcription factor, FD, forms a floral repressor complex that maintains inflorescence meristems in an indeterminate state. Arabidopsis *TFL1* is expressed only in shoot apical meristems, yet *CaTFL1a* transcripts were detected exclusively in coffee leaves. Moreover, leaf-derived *CaTFL1a* transcript retains an intron, which has not been reported for *TFL1* orthologs in other species. The ectopic expression of *CaTFL1a* in *Arabidopsis* causes extreme late flowering or prevents flowering altogether. Notably, the most severe floral repressive activity occurred in transgenic plants that spliced out the extra intron from *CaTFL1*. Yeast Two-Hybrid assays show that full-length CaTFL1a protein (fl-CaTFL1a) encoded by the fully spliced mRNA interacts with FD and *Arabidopsis* 14-3-3 protein AtGRF3, whereas truncated protein (tr-CaTFL1a) encoded by transcript that retains an intron does not interact. This evidence suggests that *CaTFL1a* may affect flowering in coffee by acting as a leaf-derived, long-distance floral repressor whose activity is controlled by alternative splicing.

## 1. Introduction

Plants endure constant challenges to maintain optimal vegetative and reproductive growth due to seasonal variations and changes in climate. Whereas successful reproduction is crucial for the propagation and evolution of plant species, sufficient vegetative biomass is also important for plant survival. Many regulatory networks help plants respond to varying conditions such as temperature, water stress, disease, insect damage, photoperiod, and other environmental factors to balance these two phases and optimize growth.

In annual angiosperms, such as the model plant *Arabidopsis thaliana*, the transition from vegetative to reproductive growth is strongly affected by day length (photoperiod) [1,2]. The transition from vegetative to reproductive growth occurs when the shoot apical meristem (SAM) produces inflorescence meristems (IM) from which determinate floral meristems (FM) form, giving rise to flowers. The maintenance of indeterminate IMs and the production of determinate FMs specify the reproductive architecture of flowering plants, including lateral inflorescence branches. The balance of inflorescence branch formation and the number of FM that produce terminal flowers is regulated by the interplay of members of the phosphatidylethanolamine-binding protein (PEBP) super family with opposing activities [3]. Key PEBP proteins in *Arabidopsis* are encoded by *TFL1* and *FLOWERING LOCUS T* (*FT*), the latter of which encodes a florigen [4,5,6]. Together, these *PEBP* genes encode related proteins that compete to regulate the balance of indeterminate IMs to determinate FMs. Despite their antagonistic functions, these proteins are highly conserved in flowering plants, sharing numerous amino acid sequence motifs [7,8].

The floral induction and shoot architecture roles of highly conserved PEBP superfamily *FT/TFL1* genes from different plant species have been studied through heterologous expression in *Arabidopsis* [9,10]. In addition to *TFL1*, other *Arabidopsis TFL1-like* genes include the floral repressors *BROTHER* of *FT* (*BFT*) and *CENTRORADIALIS* (*CEN*), whereas floral inducers include *FT*, *MOTHER* of *FT* and *TFL1* (*MFT*), and *TWIN SISTER* of *FT* (*TSF*) [11,12,13]. *Arabidopsis TFL1* is expressed in inflorescence meristems, where it is required to maintain an indeterminate state. Competition between TFL1 and FT for interaction with bZIP transcription factor FD specifies the level of indeterminacy [14,15]. The floral repressor complex (FRC) includes interacting TFL1, FD, and 14-3-3 proteins, whereas the floral activation complex (FAC) forms when FT interacts with FD and 14-3-3 proteins. The FAC irreversibly promotes the expression of flower identity genes such as *APETALA 1* (*AP1*), *LEAFY* (*LFY*), and *FRUITFULL* (*FUL*) to form determinate flowers [5,16]. Overall, a complex network of interacting transcription factors determine plant floral timing and branch architecture [17,18].

In annual flowering species, the differentiation of vegetative meristems to reproductive meristems in the same year heralds a shift to the end of their life cycle. Conversely, polycarpic perennial species must maintain vegetative growth concomitantly with flower induction year after year, suggesting more complex control of meristem identity [19]. The role of *TFL1* in annual species is well documented in *Arabidopsis* [8]; however, much less is known about *TFL1* orthologs in perennial species [20,21,22]. In perennials, the underlying mechanisms that coordinate the formation of determinate and indeterminate SAMs and axillary meristems (AM) and the transition from vegetative to reproductive development require further study.

A better understanding of the reproductive/vegetative balance in perennial plants may shed light on how asynchronous flowering is regulated. Asynchronous flowering in perennials, such as *Coffea* sp L. (coffee), results in uneven fruit maturation, which affects fruit harvest and, ultimately, beverage quality. *C. arabica* is an allopolyploid that originated from interspecific crossing between *C. canephora* and *C. eugenioides* [23,24]. Coffee plants form a central stalk with lateral-growing (plagiotropic) and upward-growing (orthotropic) branches that give rise to FMs derived via axillary meristem (AM) differentiation. The dispersal of floral buds results in the distribution of coffee fruit all along the branch [25].

Given the conserved role of *TFL1*-related genes in controlling shoot architecture and branch patterning in diverse plant species, we investigated a coffee *TFL1* homolog, *CaTFL1*, through in silico analysis, heterologous expression in *Arabidopsis*, and protein interactions. In line with our hypothesis that coffee contains genes analogous to those in *Arabidopsis* that regulate flowering and architecture, our findings suggest that CaTFL1a is a candidate component of the FRC that regulates inflorescence architecture and flowering time in coffee. Unexpectedly, we discovered that leaf-derived *CaTFL1a* transcripts have fully spliced and alternatively spliced variants. The fully spliced *s-CaTFL1a* mRNA encodes an active full-length repressor protein (fl-CaTFL1), whereas the alternatively spliced *as-CaTFL1a* version generates a truncated protein isoform (tr-CaTFL1) that lacks repressive activity. These findings suggest a post-transcriptional control mechanism for the CaTFL1a regulation of floral induction and shoot architecture in coffee.

## 2. Materials and Methods

### 2.1. Plant Material

Three biological replications of leaf samples were collected from two *Coffea arabica* cultivars, Acauã and IPAR, and one *Coffea canephora* cultivar, Conilon. Each replication was represented by a single plant, and leaves were collected approximately from the same location on the plant, specifically from the branch at the third medium part; samples for each biological replication were taken from that same plant at the defined time points. All sampled plants were four years old at the same growth stages and grown at the Federal University of Lavras experimental field, Lavras, Minas Gerais, Brazil (21°23′ S, 44°97′ W). Samples were collected in different reproductive and vegetative stages during the years 2016 and 2017 before the coffee flower induction period (DEC, 2016), at the start of flower induction (FEB, 2017), at the flower bud development (APR, 2017), at flowering time (JUN, 2017), and after flowering (OCT, 2017). These time points were chosen to represent all the reproductive stages of the coffee plant along a one-year developmental cycle.

### 2.2. In Silico Gene Identification Analysis

To identify the putative coffee, *TFL1*, we performed local alignments with Blast 2.13.0 [26] on *C. arabica* and *C. eugeniodes* sequences in the National Center for Biotechnology Information [27] and *C. canephora* sequences in the Coffee Genome Hub [28]. *Arabidopsis thaliana TFL1* (TAIR locus AT5G03840) was used as query [29]. All sequences with similarity above 80% and E-value below 0.005 were kept and globally aligned to other TFL1-like, FT-like, and MFT-like proteins from *Solanum lycopersicum* and *Arabidopsis thaliana* with CLUSTALW [30]. A relaxed E-value threshold of 0.005 was applied to capture distant homologs. The sequences were further validated by identity, query coverage, and manual inspection. With the transduced nucleotide sequence to protein, evolutionary trees were inferred with the nearest neighbor-joining method in MEGA-X 10.2 [31].

### 2.3. Gene Isolation

RNA was isolated from coffee leaves and meristem-enriched vegetative shoots of *C. arabica* cultivar Acauã collected in February 2017, following Invitrogen’s Carlsbad, CA, USA Concert™ Plant RNA Reagent organic extraction protocol (Invitrogen, Carlsbad, CA, USA). Final concentration and purity were accessed with spectrophotometric analysis (GE NanoVue™ Spectrophotometer). Samples were DNAse treated and cDNA synthetized using a High-Capacity cDNA Reverse Transcriptase Kit (Thermo Fisher, St. Louis, USA). PCR primers used for *CaTFL1a* isolation were designed and analyzed using an OligoAnalyzer™ Tool ((available at https://www.idtdna.com/page, accessed on 3 July 2026). A forward primer (5′-ATGTCGAGGCTCCTGGAA) targeting the start codon (ATG) and a reverse primer (5′-TCATCTTCTTCTTGCTGCTGTT) targeting the stop codon (TGA) were designed from the C. arabica CaTFL1a coding sequence on chromosome 2, which was selected after alignment and evolutionary analysis. Polymerase Chain Reaction (PCR) was used for gene amplification with iProof High-Fidelity DNA Polymerase (Bio-Rad, Hercules, USA) and a fragment purified from 1% agarose gel after electrophoresis run (100 v, 30 min.) with the GeneJET Gel Extraction Kit (Thermo Fisher, St. Louis, USA).

### 2.4. Expression Vector Construction and Plant Transformation

*CaTFL1a* mRNA was isolated from coffee leaves, cloned, and transformed using the Gateway system (Thermo Fisher, St. Louis, USA). Insertion tags were added to the forward and reverse primers (Fw tag—GGGGACAAGTTTGTACAAAAAAGCAGGCTAT; Rv tag—GGGACCACTTTGTACAAGAAAGCTGGGTA) and inserted into pDONR™221 with the BP Clonase enzyme. Next, the putative *CaTFL1a* construct was transformed into competent DH5α cells and confirmed through Sanger sequencing. *CaTFL1a* was then transferred from pDONR221 to the destination binary vector pK2WG7 through recombination using LR Clonase™ (Invitrogen, Burlington, Canada) according to the manufacturer’s instructions, followed by *Agrobacterium tumefaciens* transformation. *Arabidopsis* plants were cultivated in a growth chamber under long day (LD) conditions of 16 h light/8 h dark, with a temperature of 22 °C and 60% relative humidity. Heterologous expression analysis was conducted using the model plant *Arabidopsis thaliana* ecotype Columbia (Col-0). Wild-type and *TFL1* loss-of-function mutants (*tfl1-14*) were transformed by the insertion of *CaTFL1a* through *Agrobacterium tumefaciens* (strain GV3101::pMP90) infection using the overexpression construct with *Cauliflower mosaic virus p35S* promoter through the floral dip protocol [32]. Positive transformations were confirmed on ½ MS media containing 30 mg/L kanamycin, followed by DNA extraction and PCR. With PCR, we confirmed the presence and orientation of the transgene in the T1 plants. For some lines, phenotypic data at the T2 generation could not be obtained because the T1 plants continued vegetative growth and did not produce seeds.

Sequences of *tr-CaTFL1a* and *s-CaTFL1a* ORFs were each cloned into pK2GW7 vectors using the Gateway system (Invitrogen, Burlington, Canada). The *tr-CaTFL1a* and *s-CaTFL1a* ORFs were first amplified from vectors using sequence-specific PCR primers flanked by *att*B recombination sequences on the 5′ ends (Table S1). PCR products were purified using the GeneJET PCR Purification Kit (Thermo Fischer, St. Louis, USA) and inserted into binary vectors by Gateway cloning as indicated. Identity of plasmids was confirmed following colony PCR and Sanger sequencing.

### 2.5. Yeast Two-Hybrid Assay

Protein–protein interaction analysis was carried out with a Y2H assay using the Matchmaker Gold System [33]. *CaTFL1a* sequences, *s-CaTFL1a* and *tr-CaTFL1*, were amplified with iProof High-Fidelity DNA Polymerase (Bio-Rad, Hercules, USA) and gene-specific primers with an EcoRI restriction site in the forward primer and BamHI site in the reverse primer (Table S1). PCR products were purified and inserted into pBridge “bait” vectors digested with EcoRI and BamHI according to the manufacturer’s instructions (Thermo Fischer, St. Louis, USA). Digestion products were purified as previously described, and *CaTFL1a* inserts were ligated into pBridge in-frame with the DNA-binding domain (DBD). Cloning success was verified via Sanger sequencing. pBridge-*CaTFL1a* constructs were co-transformed into Gold-strain yeast with activation domain (AD)-containing pGADT7-AD (empty), pGADT7-AtFD, and pGADT7-GRF3, following Chien et al. [33]. Dilutions were plated on a synthetic defined medium (SD) lacking leucine and tryptophan (SD/-LT) to confirm co-transformation, as well as on SD medium further lacking histidine and including 5-bromo-4-chloro-3-indolyl alpha-D-galactopyranoside (SD/-LTH X∝Gal) to gauge interactions. A five-point serial dilution was prepared by taking 40 ul of yeast mix into 160 ul of sterile ddH2O. Prior to plating, OD_600_ measurements were recorded at 0.5, 0.1, 0.02, 0.004, 0.0008, and 0.00016.

### 2.6. RNAseq Analysis

Pre-processed libraries were available in the Sequence Read Archive (SRA) from the National Center to Biotechnology Information (NCBI) under BioProject ID PRJNA609253. After quality analysis using trimmomatic v.0.33 [34] with specific trimming parameters (ILLUMINACLIP:./adapters:3:25:6 SLIDINGWINDOW:4:28 MINLEN:30), approximately 183 million sequenced paired-end reads were used for alignment against the *Coffea canephora* genome DH200-94 v1 1.0 (available at https://coffee-genome-hub.southgreen.fr/ (accessed on 3 July 2026)) using the STAR v. 2.5.3a [35] aligner with default parameters. Libraries were sorted and indexed with the Samtools v.1.10 software [36] and visualized with the Interactive Genome View (IGV) 2.6 [37]. We compared libraries from *Coffea arabica* leafy samples against the *C. arabica* genome available at NCBI (GCF_003713225.1) and libraries from *Coffea Canephora* leafy samples against the *C. canephora* genome DH200-94 v1 1.0 available in the Coffee genome Hub database (available at https://coffee-genome-hub.southgreen.fr/ (accessed on 3 July 2026)). Two distinct genomes were compared to more clearly illustrate variation in the gene region, given that we were analyzing samples from two species.

## 3. Results

### 3.1. Identification of a TFL1 Homolog in Coffea sp.

**Figure 1:**
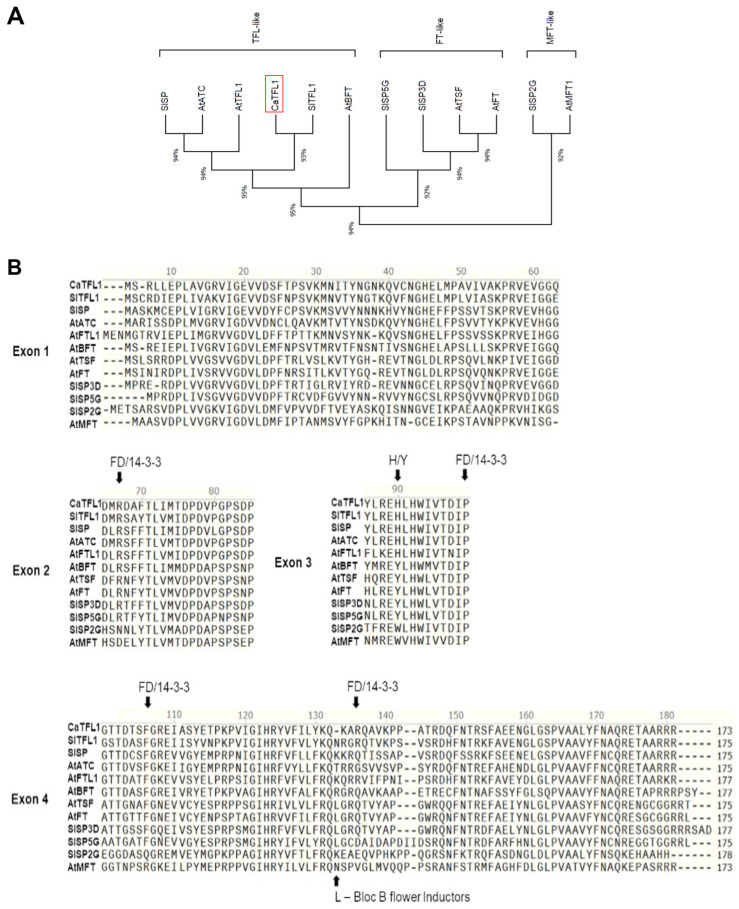
CaTFL1a amino acid sequence aligned with PEBPs from *Arabidopsis thaliana* (*At*) and *Solanum lycopersicum* (*Sl*). (**A**) Evolutionary tree showing that deduced CaTFL1a protein clades with TFL-like proteins were derived from three main PEBP flower control genes, *TFL-like*, *FT-like*, and *MFT-like*. Red box indicate selected putative coffee TFL1 protein. (**B**) *At* and *Sl* PEBP amino acid sequence alignment with CaTFL1a sequence showing amino acids likely required for FD and 14-3-3 complex formation in each exon-encoded sequence, and H/Y (histidine/tyrosine) amino-acid substitution responsible for TFL1/FT function. Arrows indicate key amino acids associated to FD/14-3-3 complex formation, and H/Y substitution.

Isolation and local alignment of *PEBP* gene sequences from public coffee sequence databases revealed several homologs with close similarity to *TFL1*-like genes from other plant species (Figure 1A and Figure S1). Multiple global alignments and trees of these sequences revealed putative *C. arabica TFL1* genes based on similarity with known protein sequences in *Arabidopsis thaliana* and *Solanum lycopersicum* that have well-established roles in regulating flowering and shoot architecture [38]. We identified three putative *TFL1*-related genes in *C. arabica* genome, one from each parental line (c—*C. Canephora* and e—*C. eugeniodes*), and putatively duplicated loci on chromosomes 1 (ch1), ch2, and ch6 (3 × 2), indicating that each putative *CaTFL1* is duplicated in both sub genomes A and B of *C. arabica*. Since these sequences have amino acid identities above 93% (Figure S1), we selected the *C. arabica* ch2 sequence, designated *CaTFL1a*, for further analysis. The similarity of this putative coffee *TFL1* ortholog to other *TFL1*-like *PEBP* genes (Figure 1A) suggests that it might be involved in flowering repression rather than floral induction [39]. Also, CaTFL1a contains conserved amino acids, such as residues R63, P95, F102, and R132 and H/Y85, which have been shown previously to mediate FRC–FAC docking via the 14-3-3 protein (Figure 1B).

### 3.2. CaTFL1a Transcript Isolated from Coffee Leaves Retains an Intron

**Figure 2:**
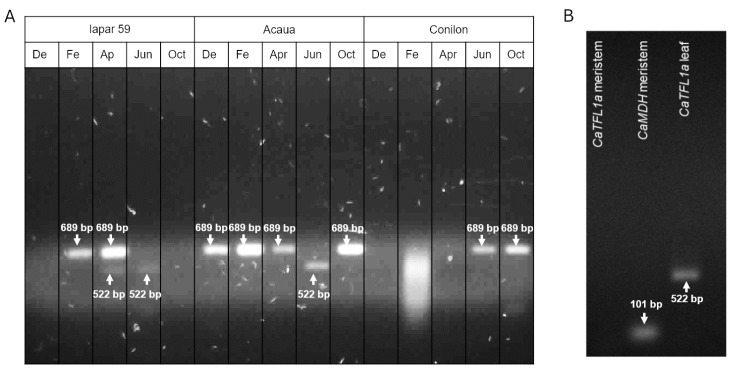
(**A**) *CaTFL1a* transcript amplified from two *Coffea arabica* genotypes (Iapar 59 and Acauã) and one *Coffea canephora* genotype (Conilon) at five different times of the year (De—December, Fe—February, Ap—April, Jun—June, and Oct—October). Arrows indicate putative spliced transcripts. Each time point is the PCR result from a cDNA pull of three biological replications. (**B**) Analysis of expression in meristematic and leaf tissue with primers for *CaTFL1a* and *CaMDH* (*C. arabica* malate dehydrogenase as positive control), showing *CaTFL1a* was not detected in meristem.

In *Arabidopsis*, *TFL1* mRNA is expressed and translated in the SAM early in development, where it competes with FT for interaction with the FD/14-3-3 complex [5,13]. However, the *CaTFL1a* transcript examined here was isolated and amplified from coffee leaves, where it is predominantly expressed (Figure 2A), and it was not detected in meristematic tissue (Figure 2B). The gene structures of *Arabidopsis TFL1* and *CaTFL1a* are similar, with each having four exons and three introns (Figure S2A,B). Unexpectedly, all mature *CaTFL1a* mRNA sequences isolated as cDNAs from coffee leaves retained the third intron, whereas the other two introns were spliced out, in contrast to orthologous *TFL1* genes from *Arabidopsis* and other species, where all three introns are spliced out (Figure S2). The amplified fragment was 689 bp, of which 522 bp corresponded to the deduced fully spliced *CaTFL1a* sequence. The remaining 167 bp corresponded to intron 3 retained in the mature mRNA. Only the 689 bp fragment generated by RT-PCR from coffee leaves cDNA was detected (Figure S2), suggesting that this alternatively spliced version is the most abundant transcript in leaf tissue. The transcript retaining intron 3, designated *as-CaTFL1a*, has a premature stop codon that results in a 125-amino-acid truncated protein, tr-CaTFL1a. The completely spliced transcript, designated *s-CaTFL1a*, encodes a 173-amino-acid protein, fl-CaTFL1a, which is highly similar to TFL1 proteins from other species.

Since these leaf samples were taken at the same time of year, in spring (April), we examined whether different splice variants were found at different times of the year. Transcripts from two Brazilian-grown *C. arabica* genotypes, Iapar 59 and Acaua, were amplified from leaf samples taken in December, February, April, June, and October. In Brazil, December has the longest days (~13.5 h), while June has the shortest days (~9 h). RT-PCR showed that the larger, alternatively spliced 689 bp transcript cDNA was the only product detected in most months in both genotypes (Figure 2). However, the fully spliced version (522 bp) of *CaTFL1a*, corresponding to *s-CaTFL1a*, was the main transcript detected in leaf tissue taken in June, or winter in Brazil (Figure 2). Moreover, transcript levels from June samples were lower relative to *as-CaTFL1a* transcripts from other times of the year. It should be noted that in the coffee genotype Conilon, only *as*-*CaTFL1a* transcripts were detected, and only in June and October (Figure 2), implying a different mode of *CaTFL1a* splicing in the Conilon branches sampled.

**Figure 3:**
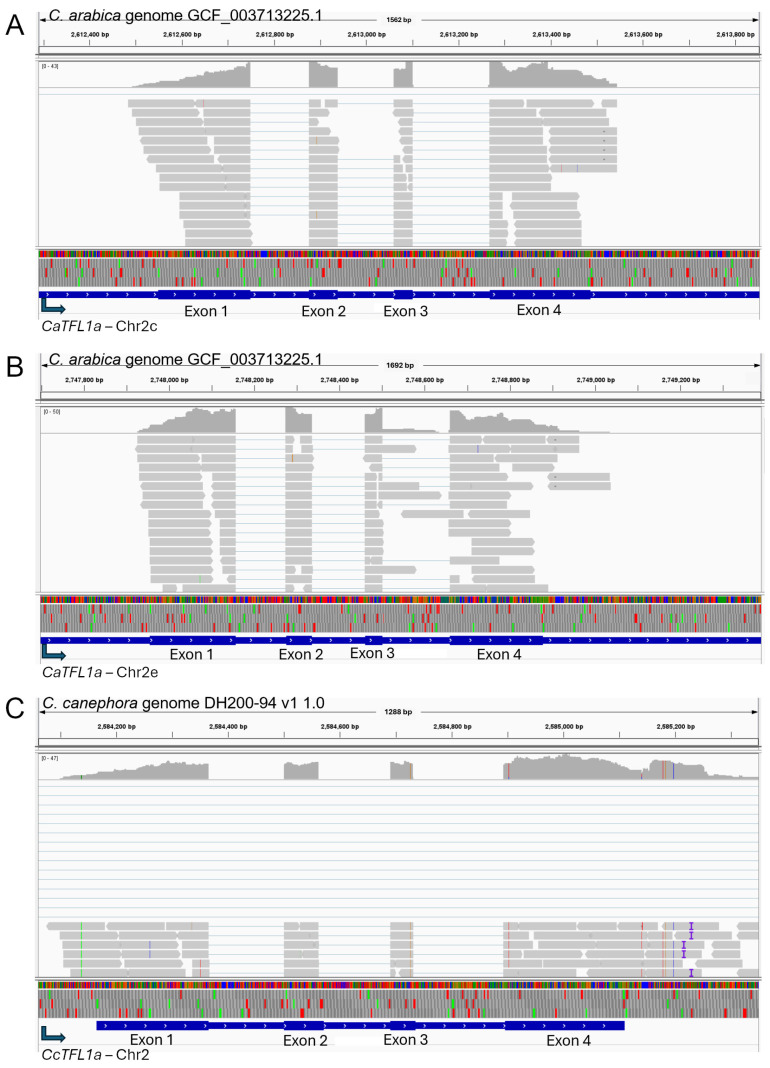
Interactive Genome Viewer (IGV) depiction of intron retention in mature *CaTFL1a* mRNA in *Coffea eugenioides* and *Coffea arabica* sub genomes based on RNAseq alignment from *C. arabica* leaf samples. Gray bars indicate reads aligned with the genome, blue bars represent the *CaTFL1a* mRNA sequence divided into four exon blocs. (**A**) *CaTFL1a* alignment in *Coffea canephora* chromosome 2 subversion sequence in *Coffea arabica* genome. (**B**) *CaTFL1a* alignment in *Coffea eugenioides* chromosome 2 subversion sequence in *Coffea arabica* genome. (**C**) *CcTFL1* alignment in *Coffea canephora* genome with transcripts from *Coffea canephora* leafy sample libraries.

To further examine *CaTFL1a* leaf transcripts, we performed an alignment with sequences from RNAseq libraries from coffee leaves available in the NCBI database and compared the *C. arabica* and *C. canephora* genomes (Figure 3). Interestingly, the aligned reads against the coffee genome showed that the alternatively spliced transcript version was detected only in the *C. eugenioides* subgenome *CaTFL1a* copy within the *C. arabica* genome (Figure 3A), with no reads aligning to the retained intron over the *C. canephora* sub-genome (Figure 3B). The same was observed for reads from *C. canephora* leaf *CaTFL1a* transcripts aligned against the *C. canephora* genome, in which no intron regions were retained in the mature transcripts (Figure 3C).

### 3.3. Overexpression of as-CaTFL1a in Arabidopsis Causes Late Flowering and Excessive Branching

**Figure 4:**
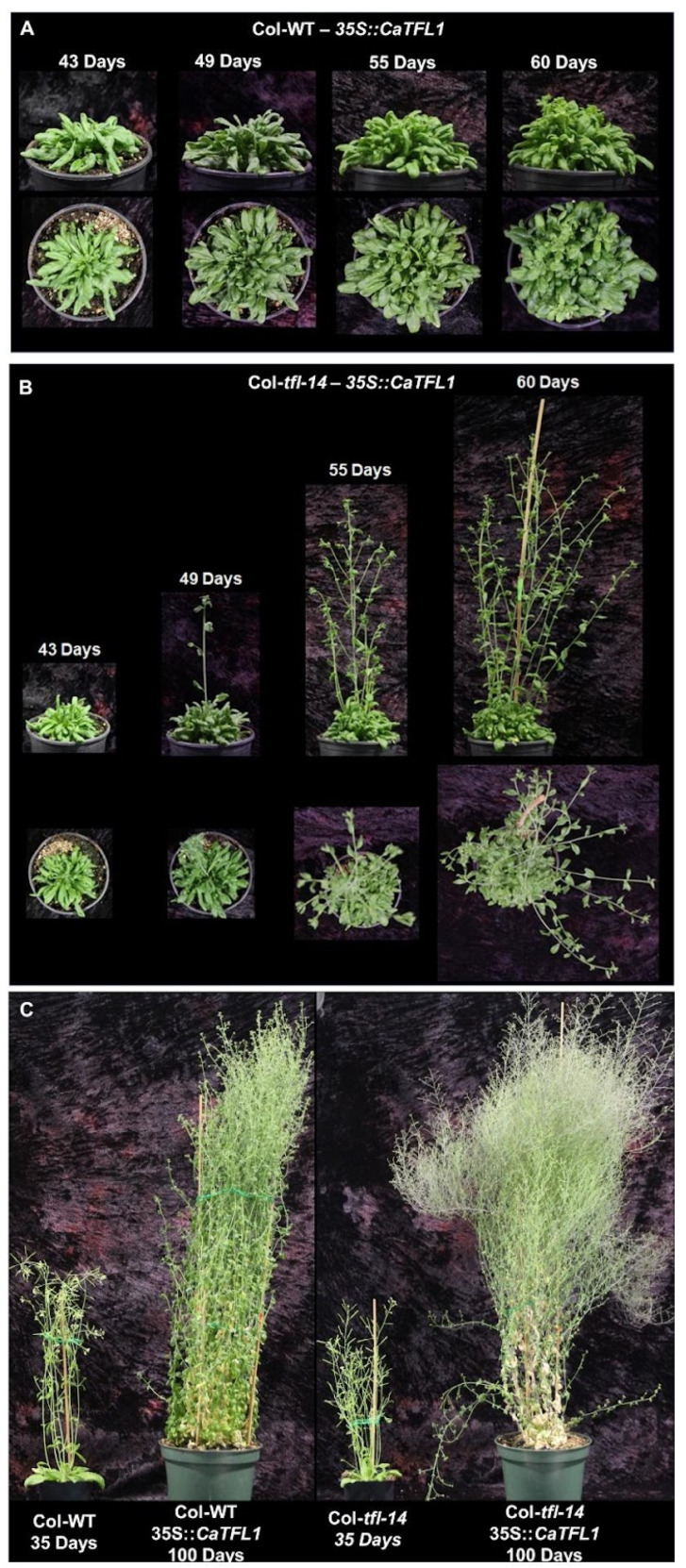
Ectopic overexpression of as-*CaTFL1a* in transgenic *Arabidopsis thaliana* Col-0 WT and *tfl1-14* loss of function mutant demonstrates late flowering and abnormal branching behavior. (**A**) *35S::as-CaTFL1a* in Col-0 showing rosette leaf formation at 43, 49, 55, and 60 days after sowing. (**B**) *35S::as-CaTFL1a* in *tfl1-14* mutant rosette leaves and IM development at 43, 49, 55, and 60 days after sowing. (**C**) From left to right: Col-0 with flower and silique formation 35 days after sowing; *35S::as-CaTFL1a* in Col-0 100 days after sowing shows extensive branching and no flowers; *tfl1-14* with terminal flower formation 35 days after sowing; *35S::as-CaTFL1a* in *tfl1-14* 10 days after floral initiation and 100 days after sowing, demonstrating numerous branches and increased IM (inflorescence meristem) development.

The *as-CaTFL1a* cDNA isolated from coffee, which retained intron 3, was overexpressed in *Arabidopsis* (Col-0) under the control of the *CaMV 35S* promoter to test its effect on flowering and plant architecture. All first-generation (T1) *CaTFL1a* overexpressing transgenic *Arabidopsis* lines displayed strong floral repression, with increased rosette leaf number, and weak apical dominance that altered the IM branching architecture in both WT and *tfl1-14* mutant backgrounds (Figure 4). Transgenics in the *tfl1* background show that ectopic *CaTFL1a* expression could restore indeterminate growth in the mutant. Indeed, ectopic *CaTFL1a* expression caused inflorescence aberrations by altering central inflorescence branch development and increasing inflorescence branch numbers. Regarding flowering time and rosette leaf number, the overexpression of *CaTFL1a* was more severe in WT Col-0 than in the *tfl1* mutant background. A comparison of nine independent T1 lines showed that all flowered later than nine *tfl1* T1 lines with the *35S::CaTFL1a* transgene. Representative transgenic plants in the WT and mutant background are shown in Figure 4A, and 4B. The overexpression of *AtTFL1* in WT *Arabidopsis* (Col-0) also caused late flowering and increased indeterminacy [14], but not as severe as compared to WT *35S::CaTFL1*. With regard to inflorescence branching, generation of IMs from the SAM occurs later when *CaTFL1a* is overexpressed. Moreover, in the most severe *CaTFL1a* overexpression lines, the indeterminate state of the IM is enhanced, greatly interfering with the IM transition to FMs and the formation of floral structures (Figure 4C). Accordingly, in these severe lines, additional new rosette leaves formed, supporting continuous growth from the indeterminate IM. This phenotype was also observed in *35S::CaTFL1a tfl1-14* lines (Figure 4C). Only after 90 DAG did these plants start to flower, but with atypical flowers that were smaller than those of WT (Figure S3). However, the IM of plants carrying *35S::CaTFL1a* in the WT background had increased levels of branching and remained indeterminate, with no flower initiation until 150 days after sowing.

### 3.4. Spliced CaTFL1a Encodes a Protein That Interacts with Arabidopsis FD and 14-3-3 Proteins While the Unspliced Variant Encodes a Truncated Protein That Does Not Interact

**Figure 5:**
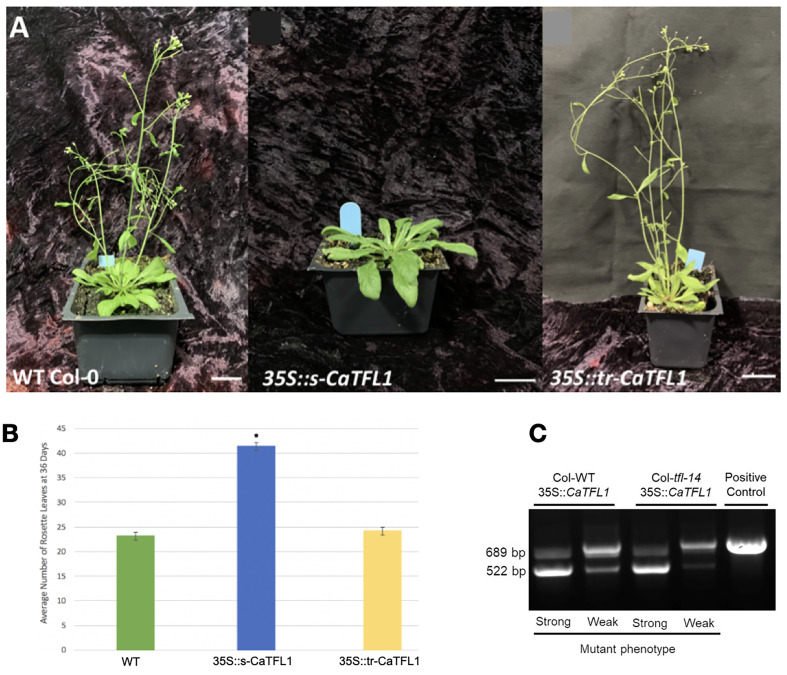
(**A**) Thirty-six-day-old WT Col-0 plants overexpressing spliced and unspliced versions of *CaTFL1*. T1 plants, left to right: WT Col-0, Col-0 *35S::s-CaTFL1a*, and Col-0 *35S::tr-CaTFL1a* grown under optimal long day conditions. At the 36-day stage, both WT (Col-0) and *35S::tr-CaTFL1a* plants had flowered and formed siliques. The T1 *35S::s-CaTFL1a* plants remained in the vegetative stage at 36 days and would continue to form rosette leaves before bolting, as shown in (**B**) with asterisks indicating approximately two-fold increase. (**C**) PCR of cDNA from leaves of *35S::as-CaTFL1a* transgenic plants (Col-WT and *tfl1-14* backgrounds) showing strong (late flowering, increased branching) and weak (slightly late flowering, normal architecture) phenotypes. Spliced (lower band, 522 bp) and unspliced (upper band, 689 bp) versions of *CaTFL1a* are from strong and weak phenotype lines, respectively. Scale bars = 3 cm.

*Arabidopsis* transformed with the *as-CaTFL1a* gene showed a wide range of phenotypes, ranging from very late flowering with numerous branches to only moderately late flowering (Figure 5A,B). Given that *as-CaTFL1a* retains intron 3, we examined the transcripts from both strong and weak phenotypes and found that plants with the most severe phenotypes had a large proportion of transcripts that had spliced out intron 3, whereas the unspliced transcript predominated in weaker phenotypes (Figure 5C). This shows that splicing of this coffee transcript in *Arabidopsis* occurs stochastically and correlates with the severity of the architecture and flowering phenotype. The spliced sequence, *s-CaTFL1a*, encodes a deduced full-length protein, designated fl-CaTFL1a, which shows 100% similarity with the deduced *CaTFL1a* sequences from the *C. arabica*. However, due to an in-frame stop codon, the retained intron in *as-CaTFL1a* encodes a truncated protein, designated tr-CaTFL1a (Figure S2).

**Figure 6:**
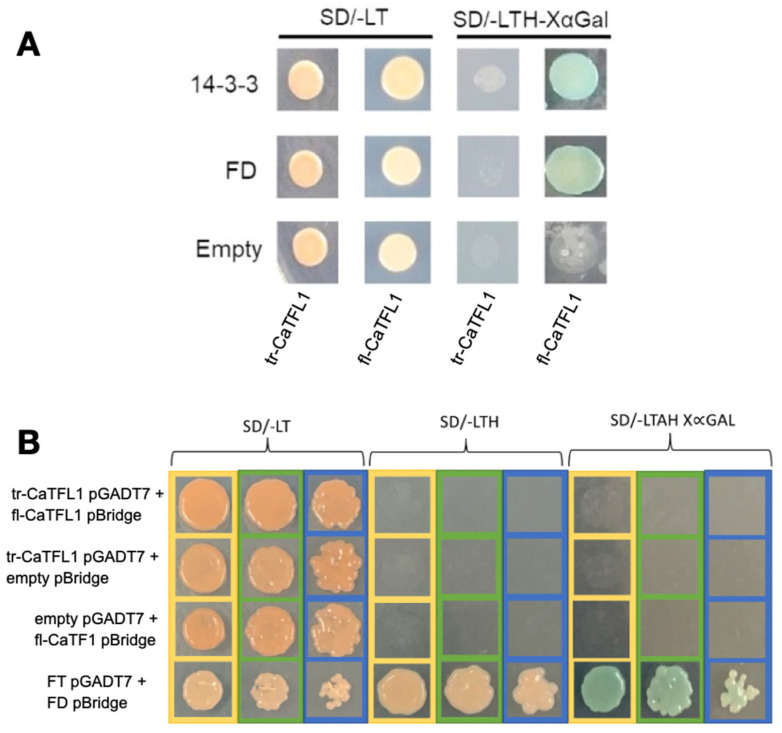
(**A**) Yeast Two-Hybrid assay with *as-CaTFL1a* and *s-CaTFL1a* sequences encoding tr-CaTFL1a (truncated) and fl-CaTFL1a (full-length) proteins. Left side shows prey constructs, AtGRF3 (14-3-3), AtFD, and empty plasmid. Below indicates bait constructs *tr-CaTFL1a* and *fl-CaTFL1*; plated on non-selective SD/-LT and selective SD/-LTH XαGal media. Five-time serial dilution was used at OD600 = 0.5, 0.1, 0.02, 0.004, 0.0008, and 0.00016. (**B**) Y2H analysis of whether full-length (fl-CaTFL1) or truncated (tr-CaTFL1) proteins can form a heterodimer. *Arabidopsis* FT and FD proteins are positive controls for interaction.

To test the activities of these proteins, Yeast Two-Hybrid (Y2H) analysis was used to determine whether they could interact with the *Arabidopsis* FD protein, similar to AtTFL1. The floral repressor complex (FRC) additionally contains a 14-3-3 protein which is believed to bridge interactions between PEBPs and FD. Therefore, we tested the interaction with an *Arabidopsis* 14-3-3 protein encoded by the *Arabidopsis GRF3* gene (Figure 6). We found that the fl-CaTFL1a protein translated from the fully spliced sequence interacts with *Arabidopsis* FD and 14-3-3 (Figure 6A). By contrast, the truncated tr-CaTFL1a protein does not interact with FD or 14-3-3. These findings support the transgenic analysis, which showed that the ectopic expression of *tr-CaTFL1a* does not affect *Arabidopsis* architecture or floral development, whereas the overexpression of *s-CaTFL1a* has a severe late-flowering effect and altered branching (Figure 5A,B). At 36-days post-germination (DPG), WT and *35S::tr-CaTFL1a* plants possessed normal inflorescences and had flowered similar to WT (Figure 5B). In contrast, *35S::s-CaTFL1a* transformant plants remained in a vegetative stage at 36 DPG and lacked reproductive structures. WT and *35S::tr-CaTFL1a* plants bolted at an average of 27.33 (*n* = 3) and 28.33 (*n* = 3) days, respectively, showing almost the same value for both. In contrast, *35S::s-CaTFL1a* plants bolted, on average, about 47.5 days post-germination (*n* = 2), which was approximately two-fold in comparison to WT plants. Accordingly, the average number of vegetative rosette leaves of WT and transformant plants at the 36 DPG stage differed (Figure 5B). WT plants had an average of 23.3 rosette leaves (*n* = 3), whereas *35S::tr-CaTFL1a* plants had an average of 24.3 (*n* = 3), a very close result. As for *35S::s-CaTFL1a*, the plants also had approximately two-fold more rosette leaves than WT plants, with an average of 41.5 (*n* = 2).

### 3.5. Full-Length fl-CaTFL1a Protein Does Not Interact with the Truncated tr-CaTFL1a Protein

Truncated protein isoforms have been reported to regulate full-length isoforms in plants, such as by forming heterodimers incapable of generating FAC or FRC complexes [40]. Such is the case for FT2 of *Brachypodium distachyon*, a FT homolog [41]. *FT2* undergoes an alternative splicing event that generates a truncated protein isoform missing the N-terminal portion of its PEBP domain. Like tr-CaTFL1a, truncated FT2-β isoforms lack the ability to interact with FD/14-3-3, whereas the fully spliced FT2-α isoform can interact with both proteins. Y2H evidence suggests that FT2-α and FT2-β form heterodimers, preventing FT2-α from forming a FAC. Another possible means of regulation via alternative splicing involves the formation of heterodimers with decreased stability. This has been observed in heterodimers formed between the full-length and truncated isoforms of CONSTANS (CO) [42]. When the truncated CO isoform interacts with the full-length isoform, the full-length isoform is ubiquitinated and targeted for degradation by the 26S proteasome. Given these possibilities, we found that CaTFL1a protein isoforms do not appear to possess the ability to form heterodimers in yeast since co-transformed tr-CaTFL1a-AD and s-CaTFL1a-DBD yeast did not grow on selective media following a 3-day incubation at 30 °C (Figure 6B). In contrast, positive controls of yeast co-transformed with the known interactors FT and FD showed growth in both forms of the selective media. Two negative controls featuring both *CaTFL1a* variant constructs co-transformed with empty vectors did not grow on the selective media, as expected (Figure 6B). Taken together, these Y2H results suggest that the fl-CaTFL1a and tr-CaTFL1a variant proteins do not form heterodimers.

## 4. Discussion

The basic molecular elements of floral induction are shared by diverse plants, ranging from herbaceous annuals to woody perennial species, such as coffee. Conserved components include PEBP family proteins such as a leaf-derived florigen, typified by the *Arabidopsis* FT protein, and its homologous counterpart, TFL1, which acts antagonistically to FT to maintain an indeterminate state [3]. In a previous study, we identified a coffee florigen gene, *CaFT1*, and showed that it can rescue the late-flowering defect of the *Arabidopsis ft* mutant, suggesting that it has a similar role in coffee [43]. Here, we have identified *CaTFL1a*, a homolog of *AtTFL1* that has similar properties of maintaining the *Arabidopsis* inflorescence meristem in an indeterminate state, as well as prolonging the vegetative state. The expression of coffee *CaTFL1a* in *Arabidopsis*, a heterologous plant, has a similar effect as the overexpression of the endogenous *TFL* gene; that is, late flowering and increased branching. This is a first step in understanding how *CaTFL1a* may function in coffee. Further research is required to determine the precise role *CaTFL1a* plays in regulating coffee flowering and branch architecture.

Comparison of the deduced CaTFL1a amino acid sequence with PEBPs from other species shows strong evidence that the sequence isolated from *Coffea* is a floral repressor similar to TFL1. Variations of these sequences that have been shown to be important for delineating TFL1 and FT function further support CaTFL1a as a floral repressor [16,44,45]. Overall, these similarities suggest that CaTFL1a acts as a floral repressor with a role in maintaining reproductive meristem indeterminacy in *Coffea*. Moreover, we show that several features of CaTFL1a suggest that it controls coffee flowering time and the inflorescence architecture in a novel way. First, CaTFL1a appears to be expressed in mature leaf tissue rather than in meristems. Second, the predominant transcript retains an intron that, if translated, would produce a truncated version of the CaTFL1a protein.

Whereas *Arabidopsis TFL1* and currently identified orthologs from other plant species are localized to the shoot apical meristem, we detected *CaTFL1a* transcripts exclusively in mature coffee leaf tissue. Leaf expression is typical of *PEBP* genes that encode florigen, a phloem-derived floral inducer. The localization of *CaTFL1a* transcript in leaves, and presumably its translation product, suggests that CaTFL1a protein may be similarly mobile and that it moves through plant vasculature to reach meristematic regions. However, in our analysis, we did not investigate whether CaTFL1a protein moves through the vasculature from leaf to apex, as this would be an exceedingly difficult experiment to conduct. However, a previous study showed that the ectopic expression of *TFL1* in *Arabidopsis* leaf primordia and other tissues altered floral architecture, suggesting that the floral inhibitor can be expressed outside the shoot apex and act at a long distance [46]. Other PEBP floral inhibitors have also been shown to be localized to plant tissues other than at the shoot apex. For example, *ATC* (*Arabidopsis thaliana CENTRORADIALIS*) and *BFT* (*BROTHER* of *FT* and *TFL1*) encode *Arabidopsis* PEBP floral repressors that are expressed outside the shoot apex in vascular tissues [12,47]. However, a phylogenetic analysis shows that CaTFL1a protein is more closely related to TFL1 than ATC or BFT.

Next, most *CaTFL1a* transcripts isolated from coffee leaves retained one of the four cognate introns. Although a completely spliced *s-CaTFL1a* version was detected in leaf samples taken at other times of the year and in different coffee accessions, the larger, alternatively spliced transcript predominates. Thus far, the presence of *as-CaTFL1a* versus *s-CaTFL1a* does not appear to be correlated with a particular photoperiodic pattern; that is, neither short days nor long days favor either splice variant consistently. However, this finding brings up the intriguing possibility that the regulation of coffee flowering time and branch architecture could be controlled by a post-transcriptional mechanism. Selective intron retention that gives rise to alternate proteins that modify their ability to interact with other proteins or to create different complexes is not uncommon [48]. In the case of *CaTFL1a*, the control of splicing seems to dictate whether a functional repressor is made. As described above, splice variation in flowering time genes, such as *CO* and *FT2*, is not unprecedented. In *Arabidopsis*, variant CO translation products form heterodimers between the truncated and full-length CO, which inhibits CO signaling [41,42]. However, we detected no heterodimerization between fl-CaTFL1a and tr-CaTFL1, suggesting that fl-CaTFL1a cannot be inhibited directly by tr-CaTFL1a interaction. Nonetheless, we have shown that fl-CaTFL1a can interact with FRC components and acts as a strong floral repressor when ectopically expressed in *Arabidopsis*. Overall, as far as we know, this is the first report of alternate *PEBP* splicing affecting floral repression in a tropical perennial.

Furthermore, the specific expression of *CaTFL1a* in mature leaves suggests another interesting possibility, that the regulation of *CaTFL1a* splicing is environmentally controlled. How the developmental phenology of coffee is dictated by environmental signals is not clearly understood. Previously, our group suggested that photoperiod may influence coffee flowering [43]. However, floral signals most likely are regulated through the interplay of the photoperiod with other stimuli, like temperature, water, and photosynthate availability. These complex interactions may contribute to the asynchronous flowering patterns typical of coffee and other tropical perennials. It will be interesting to examine whether the control of CaTFL1a activity via alternate splicing regulates coffee flowering time and shoot architecture in response to external stimuli. Furthermore, does the asynchronous production of fl-CaTFL1a in leaves underpin the prolonged flowering pattern of coffee? Better understanding how CaTFL1a functions in vivo may allow for the development of genetics with improved flowering synchronicity, facilitating uniform ripeness and a more efficient coffee harvest.

## Data Availability

The original contributions presented in this study are included in the article/Supplementary Material. Further inquiries can be directed to the corresponding authors.

## Supplementary Materials

The following supporting information can be downloaded at: https://www.mdpi.com/article/10.3390/plants15142162/s1, Figure S1: TFL1 protein sequence alignment and evolutionary tree; Figure S2: CaTFL1a splicing variation; Figure S3: Abnormal inflorescences in *Arabidopsis thaliana* transformed with *CaTFL1a*; Table S1: Primer deigns for cloning, PCR analysis and RT-qPCR analysis.
