# Supplementary material for: A Leaf-Expressed TERMINAL FLOWER1 Homolog from Coffee with Alternative Splice Forms Alters Flowering and Branching in Arabidopsis
Source: Plants (Basel). 2026 Jul 14;15(14):2162. doi: 10.3390/plants15142162 (PMC13416373; doi:10.3390/plants15142162)
Supplement: Supplementary file 1 [file plants-15-02162-s001.zip › plants-4360465-supplementary.pdf]

Supplemental material

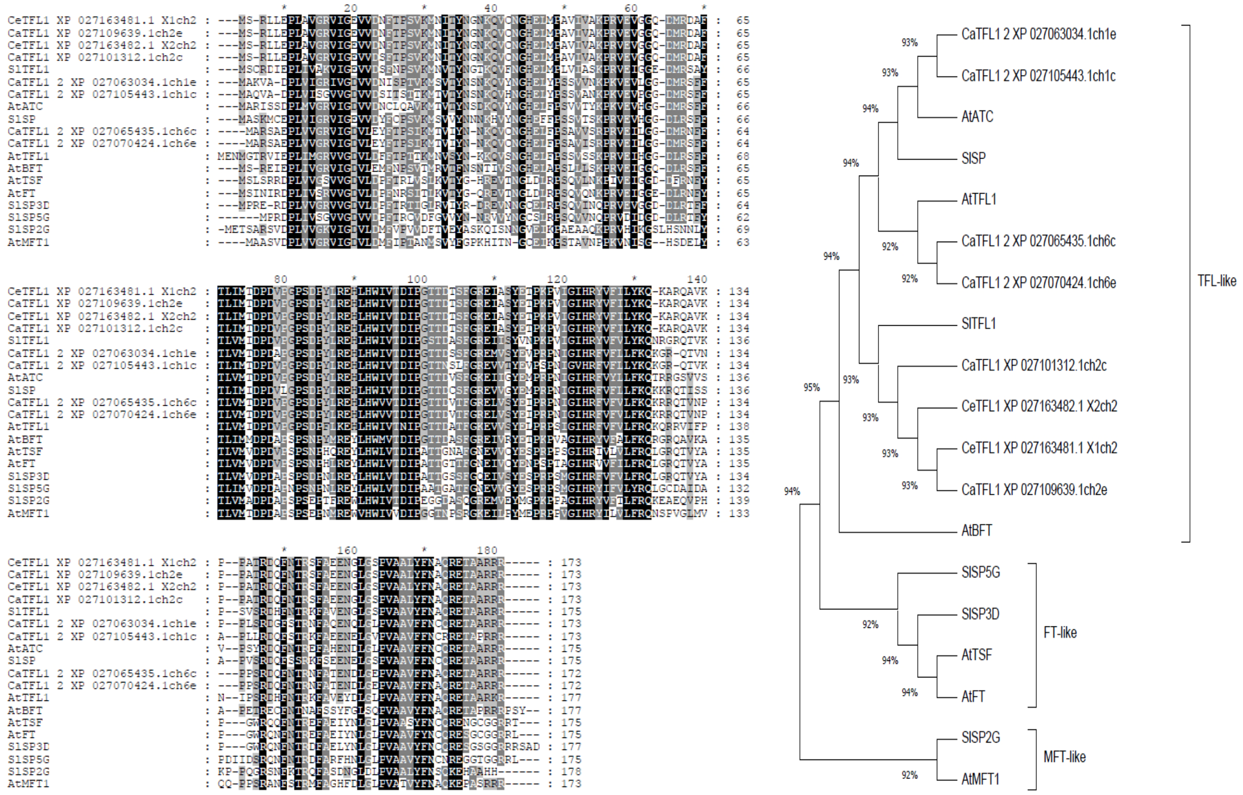

FIGURE S1. TFL1 protein sequence alignment and evolutionary tree. **A.** Putative coffee TFL protein sequences alignment against *Arabidopsis thaliana* and *Solanum lycopersicum* TFL-like, FT-like and MFT-like proteins. Sequences aligned through Genedoc tool which shows shared sequence similarity intensity from higher to lower by black, dark grey, light grey and no color, respectively. **B.** Evolutionary tree inferred by the nearest neighbour joining method in MEGA-X to separate TFL-like from FT-like and MFT-like sequences.

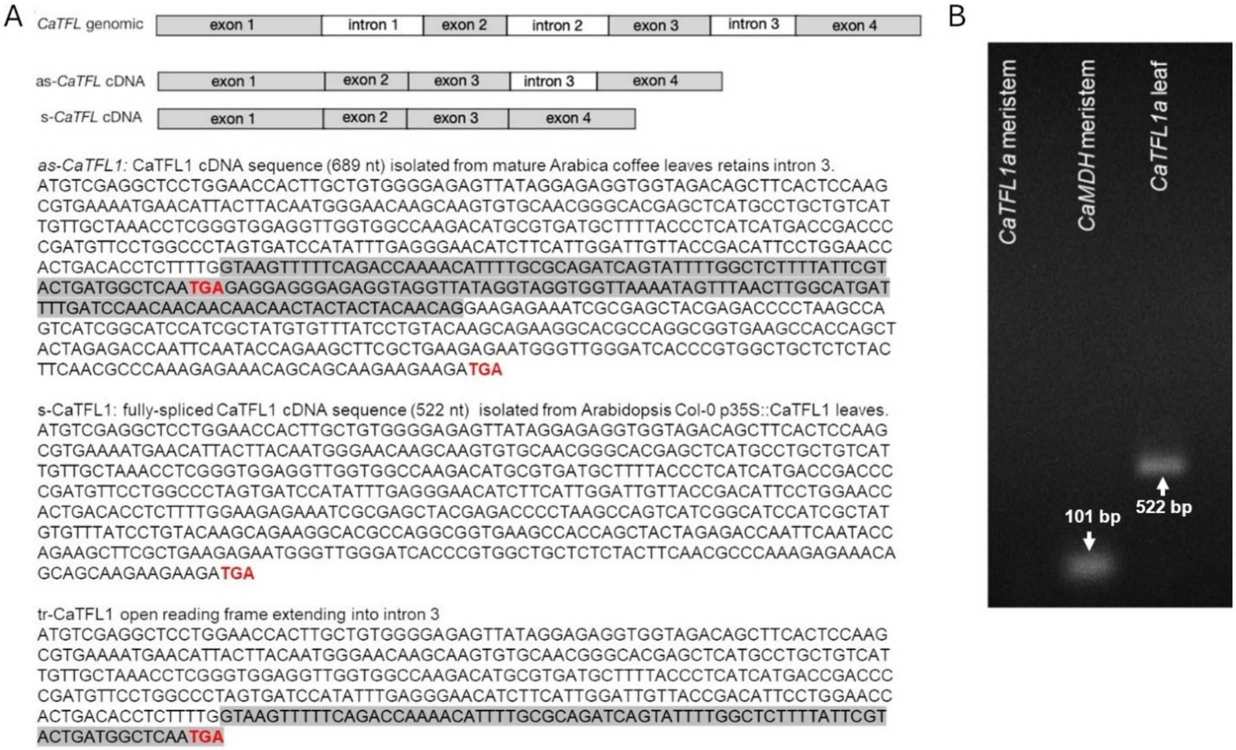

Figure S2: CaTFL1a splicing variation. **A -** TOP: Schematic of *CaTFL1a* exon-intron structure from genomic DNA and differently spliced versions; as-CaTFL1, alternately spliced with intron 3 retained, s-CaTFL1, all introns spliced out. BOTTOM: ***as-CaTFL1, s-CaTFL1 and tr-CaTFL1* sequences**. Stop codon shown in red; intron sequences highlighted in grey. **B** – PCR with primers for *CaTFL1a* amplification in coffee meristem, positive control (*CaUBQ*) on coffee meristem, and *CaTFL1a* on coffee leaf.

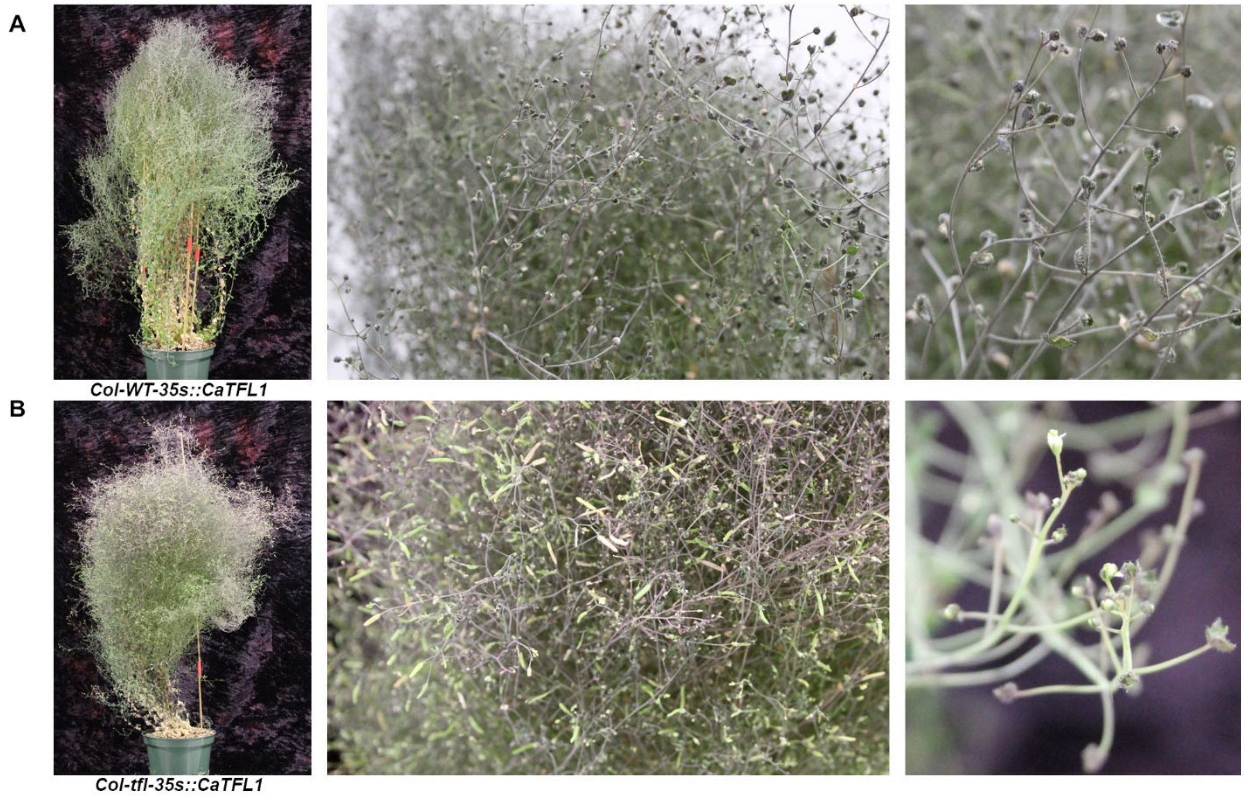

FIGURE S3 – Abnormal inflorescences in *Arabidopsis thaliana* transformed with *CaTFL1a.* **A**: T1 35S::*CaTFL1* mutant line 1 in Col-WT background 150 days after germination. **B**: T1 35S::*CaTFL1* mutant line 3 in Col-*tfl* mutant background 150 days after germination.

Table S1 –Primer deigns for cloning, PCR analysis and RT-qPCR analysis. All primers were designed using the IDT's OligoAnalyzer and NCBI's Primer-BLAST.

Purpose	Gene	Forward 5' –> 3'	Reverse 5' –> 3'
PCR/Cloning with *att*B sites	*tr-CaTFL1*	GGGGACAAGTTTGTACAAAAAAG CAGGCTATATGTCGAGGCTCCTGGA A (Yellow highlight corresponds to attB1)	GGGACCACTTTGTACAAGAAA GCTGGGTATCATTGAGCCATC AGTAC (Green highlight corresponds to *att*B2)
PCR/Cloning with *att*B sites	*s-CaTFL1*	GGGGACAAGTTTGTACAAAAAAG CAGGCTATATGTCGAGGCTCCTGGA A (Yellow highlight corresponds to *att*B1)	GGGACCACTTTGTACAAGAAA GCTGGGTATCATCTTCTTCTTG CTGCTG (Green highlight corresponds to *att*B2)
PCR/Cloning with *Nde*I and *Xho*I sites	*tr-CaTFL1*	ATACATATGATGTCGAGGCTCCTGG AA (Yellow highlight corresponds to *Nde*I)	ATATCTCGAGTCATTGAGCCA TCAGTAC (Green highlight corresponds to *Xho*I)
RT-PCR	Distinguishing between *as-CaTFL1 and s-CaTFL1* based on transcript length	ATGTCGAGGCTCCTGGAA	TCATCTTCTTCTTGCTGCTG
